# Understanding the Antecedents of Korean High School Students’ Drinking Refusal Self-Efficacy: Parental Influence, Peer Influence, and Behavior

**DOI:** 10.5539/gjhs.v4n1p10

**Published:** 2012-01-01

**Authors:** Su Ahn Jang, NamAuk Cho, Jina Yoo

**Affiliations:** Department of Communication, University of Missouri - St. Louis St. Louis, MO 63121, USA Tel: 1-314-516-5498 E-mail: jangs@umsl.edu; Department of Graduate School of Counseling Welfare & Policy, Kwangwoon University Seoul, the Republic of Korea E-mail: auk1974@hanmail.net; Department of Communication, University of Missouri - St. Louis St. Louis, MO 63121, USA E-mail: yooji@umsl.edu

**Keywords:** Drinking refusal self-efficacy, Alcohol use, Parental influence, Peer influence, Korean adolescents

## Abstract

The current study examined the factors that influence Korean adolescents’ drinking refusal self-efficacy, which is known to be associated with alcohol use and drinking intentions. Specifically, this study considered parental monitoring, parent-child communication satisfaction, peer influence, and prior alcohol use as possible antecedents of Korean high school students’ drinking refusal self-efficacy. High school students (n = 538) in South Korea responded to the current study. The data revealed that parent-child communication satisfaction facilitated parental monitoring, and these factors indirectly predicted adolescents’ drinking behavior through peer influence. We also found that prior drinking, parental monitoring, and peer influence were directly associated with drinking refusal self-efficacy, and the self-efficacy, in turn, was associated with drinking intentions. These results not only suggest that drinking refusal self-efficacy are related to drinking behavior and intentions, but they also provide a theoretical explanation for how parental and peer influences are associated with adolescents’ drinking refusal self-efficacy.

## 1. Introduction

Research suggests that alcohol is the most widely used drug by adolescents regardless of ethnicity, gender, or race (Van Der Vorst, Engels, R., Meeus, & Deković, 2006). Adolescents’ drinking behavior has been not only a problem in the U.S. (Perry *et al.*, 1996), it is also one of the vital social issues in several countries in Europe and Asia (Shin & Delva, 2004). In Korea, for instance, alcohol use among adolescents is of great concern as the average age of drinking initiation fell from 15.1 years in 1998 to 13.1 years in 2006 (Korean Ministry of Health and Welfare, 2007). This trend is alarming since scholars suggest that adolescents with early exposure to a large amount of alcohol use tend to be at greater risk for later alcohol abuse and dependence, unwanted pregnancy, suicide, domestic violence, accidents, sexually transmitted diseases, and antisocial characteristics, to name a few of the possible consequences (DuRant, Smith, Kreiter, & Krowchuk, 1999; Hingson, Heeren, & Winter, 2006). Given the problems associated with Korean adolescents’ alcohol consumption, studies identifying the ways to prevent teens from abusing alcohol are particularly important.

According to Baldwin, Oei, and Young (1993), educating adolescents on how to refuse drinking alcohol may be one approach that could decrease their alcohol use because individuals’ perceived ability to resist drinking alcohol, namely drinking refusal self-efficacy, has a vast impact on their drinking behavior. Adolescents who believe that they could resist alcohol would be more likely to refuse drinking alcohol when compared with those who lack such a perception. In fact, this approach suggests that a lack of such perception is positively associated with alcohol consumption (Oei & Jardim, 2007). Thus, theoretically, identifying the factors that affect adolescents’ drinking refusal self-efficacy and fostering the self-efficacy may facilitate decreasing adolescents’ drinking intentions and behavior.

Although numerous scholars have demonstrated the inverse association between drinking refusal self-efficacy and drinking behavior (Baldwin *et al.*, 1993; Oei & Jardim, 2007), relatively little is known about the attributes that may influence drinking refusal self-efficacy, a type of self-efficacy which ultimately influences individuals’ drinking intentions and behavior. What has been identified thus far is that parental and peer influences may be related to adolescents’ tendency to use alcohol (Hwang & Akers, 2006; Kunkel, Hummert, & Dennis, 2006; Watkins, Howard-Barr, Moore, & Werch, 2006), and drinking experience may impact individuals’ drinking refusal self-efficacy (Aas, Klepp, Laberg, & Aaro, 1995; Oei & Morawska, 2004). In an attempt to identify the attributes that predict Korean adolescents’ drinking refusal self-efficacy, a purpose of the current study was to examine how parental influence, peer influence, and drinking experience are related to Korean high school students’ drinking refusal self-efficacy.

### 1.1 Drinking Refusal Self-Efficacy

The notion of self-efficacy affecting people’s behavior is originally proposed by Bandura’s (1986) social cognitive theory. Bandura states that perceived self-efficacy plays an important role in social cognitive theory because it “supports the type of efficient analytic thinking needed to [discover] predictive knowledge from causally ambiguous environments in which many factors combine to produce effects” (p. 35). He suggests that individuals’ beliefs about the ability or capability of performing a behavior are powerful information that can predict people’s actual behavior. Self-efficacy has been examined in a variety of behaviors, including exercise (Rimal, 2000), learning (Linnenbrink & Pintrich, 2003), and communication (Afifi, & Weiner, 2004), and a strong relationship between perceptions of self-efficacy on these behaviors and actual behaviors was documented. Research also shows evidence of the connection between drinking refusal self-efficacy and drinking intentions, as well as actual consumption (Baldwin *et al.*, 1993; Watkins *et al.*, 2006). Adolescents who think that they could resist drinking alcohol would be more likely to refuse to drink when compared with adolescents who perceive a lack of such self-efficacy (Oei & Baldwin, 1994; Watkins *et al.*, 2006). In fact, the effects of drinking refusal self-efficacy on drinking behavior have been investigated in diverse samples, and a negative association between drinking refusal self-efficacy and teens’ drinking intentions and behavior with both Asian and Caucasian samples were documented (Kim, 2001; Oei & Jardim, 2007). Thus, the following hypothesis was proposed:

*H1*: Drinking refusal self-efficacy will be negatively associated with Korean adolescents’ drinking intentions.

One key predictor of drinking refusal self-efficacy is individuals’ past alcohol use. Numerous scholars support the idea that adolescents who are already using alcohol would have relatively low drinking refusal self-efficacy than those without an experience of alcohol (Aas *et al.*, 1995; Oei & Morawska, 2004). Research suggests that past behavior strongly impacts intentions and future behavior, particularly relating to habitual behaviors, such as drinking, and this relationship may be mediated by individuals’ self-efficacy perception (Aas *et al.*, 1995; Oei & Morawska, 2004). Indeed, social cognitive theory (Bandura, 1986) suggests that successful expereince is associated with individuals’ self-efficacy, which, in turn, impacts future behavior. Guided by this notion, adolescents who consume alcohol may perceive low drinking refusal self-efficacy because their successful drinking experience may reinforce their perceived ability to drink, and as a consequence, they may not have intentions to turn down alcohol offers and may continue to abuse alcohol. In a similar vein, abstinent adolescents who successfully refused alcohol proposals in the past may have relatively high drinking refusal self-efficacy when compared with alcohol users who lack experience in effectively rejecting drinks from others. Given that past experience may be linked with adolescents’ drinking refusal self-efficacy (Bandura, 1986), the following hypothesis was posed to examine the link between Korean adolescents’ prior drinking and their drinking refusal self-efficacy:

*H2*: Prior alcohol use will be negatively associated with Korean adolescents’ drinking refusal self-efficacy.

Though past drinking may be a predictor of drinking refusal self-efficacy, in order to fully understand the factors that shape adolescents’ drinking refusal self-efficacy, it would be valuable to answer the following inquiry: What is the antecedent of Koeran adolescents’ alcohol use? Identifying the factors that impact the adolescents’ drinking behavior would be helpful in fully understanding the process in which they perceive drinking refusal self-efficacy.

### 1.2 Parental and Peer Influences on Drinking

Research demonstrates that parental and peer influences emerge as the two main forces that impact adolescents’ drinking. Although some development theorists argue that parental influence tends to give way to peer influence during adolescence (Masten, Faden, Zucker, & Spear, 2008; Wood, Vinson, & Sher, 2001), other scholars continue to suggest that parental influence not only increases during late adolescence (see Duncan *et al.*, 1994), it also moderates the effects of peer influence on drinking behavior (Laird, Pettit, Bates, & Dodge, 2003;Wood, Read, Mitchell, & Brand, 2004). Because parenting and family interactions have major influences on adolescent development, behavior, and substance use (Masten *et al.*, 2008; Nash, McQueen, & Bray, 2005), the developmental model of Patterson (Patterson, DeBaryshe, & Ramsey, 1989) explains the unique dynamic between parental and peer influence as follows: Children’s delinquent behavior is due to peer influences, but children’s involvement with deviant peers is a result of poor parenting, suggesting the importance of parenting practices on children’s behavior. Similarly, Steinberg (2001) suggests that peer influence plays a role in intensifying adolescents’ delinquent behaviors, but “it is unlikely that peer influence leads to the initial emergence of these traits” (p. 12).

#### 1.2.1. Parental Monitoring

Specifically, socialization theories (Grusec & Davidov, 2010) explain that, during adolescence, parents may sense difficulty influencing their children to overcome socialization by peers, indicating the struggle between parents and peers. While some parents may underestimate the power to transmit their values to their children during adolescence, scholars emphasize that parents can protect their children from negative peer influence by monitoring their activities and whereabouts (Bogenschneider, Wu, Raffaelli, & Tsay, 1998). Consistent with the view of this theory, by restricting children’s contacts with delinquent peers, parental monitoring can prevent adolescents’ involvement in problem behaviors (Dick, Viken, Purcell, Kaprio, Pulkkinen, & Rose, 2007; Westling, Andrews, Hampson, & Peterson, 2008). For instance, Westling *et al*. suggest that little parental monitoring was related to children’s poor choice in friends, and as a result, middle and high school students committed deviant behaviors, including drinking. Likewise, Hwang and Akers (2006) demonstrate that peer influence is the mediator between parental supervision and Korean high school students’ substance use.

It is important to note that numerous scholars measure parental monitoring by the adolescents’ report of monitoring rather than the parents’ own account of their monitoring practices (Nash *et al.*, 2005; Wood *et al.*, 2004). According to Barnes and Farrell (1992), although both mothers’ and adolescents’ reports of monitoring are negatively associated with the adolescents’ alcohol use, because parents often perceive themselves as strict and providing more monitoring than what their children perceive, the use of the adolescents’ report of parental monitoring is a relatively more conservative measure when compared with using the parents’ report of monitoring. In a related vein, when assessing peer influence, studies typically include the adolescents’ own account of influence from the peers rather than asking the respondents’ friends’ to report on their influence on the respondents (Hwang & Ackers, 2006; Nash *et al.*, 2005; Wood *et al.*, 2004). For this reason, the current study adopted Korean high school students’ own account for peer influence and parental monitoring. Guided by socialization theory, the following hypothesis was posed to examine the mediating role of peer influence between parental monitoring and adolescents’ alcohol use:

*H3*: Peer influence will mediate the association between parental monitoring and Korean adolescents’ alcohol use.

#### 1.2.2 Parent-Child Communication Satisfaction

Another perspective, the individuation-connectedness, also emphasizes parental influence on children’s peer relationships (Youniss & Smollar, 1985). Youniss and Smollar suggest that, during adolescence, children make steps towards independence from parents while striving to stay connected to them, and this process that occurs in the environment of close relations with parents is optimal. If the parent-child relationship is one of interdependence and has a cooperative climate, adolescents would continue to seek parental support and allow parental influence over peer relationships (Steinberg, 2001; Youniss & Smollar, 1985). Parental responsiveness, which typically occurs during verbal interaction with children, is a correlate of children’s social competence and choice of friends (Lamborn *et al.*, 1991). In view of that, communication helps children maintain close relationships with their parents because it promotes children’s cognitive and social competence and results in parent-child satisfaction and more competence in adolescents’ interactions outside the home environment (Steinberg, 2001).

The individuation and connectedness perspective suggests that parent-child communication, rather than unilateral parental monitoring, is the way in which parents influence adolescent children’s peer relationships (Bray, Adams, Getz, & Stovall, 2001; Youniss & Smollar, 1985). Numerous scholars demonstrate that children and parents freely sharing emotional and factual information is indicative of good communication, and it may have a greater impact on children’s behavior than parental monitoring alone (Cohen & Rice, 1995; Otto & Atkinson, 1997). In fact, Cernkovich and Giordano (1987) suggest that juvenile delinquents’ home environment is short on communication about future plans or children’s problems with friends or teachers, and the lack of communication with the parents may result in greater peer influence.

While research focuses on the importance of good parent-child communication on adolescents’ peer relationships, it is relatively unclear whether knowledge and skills learned during communication *or* whether the satisfaction children feel from communicating with their parents is the drive behind for adolescents’ behavior outside the home. Barbato, Graham, and Perse (2003) suggest that parents’ primary motive for communication with their children is affection. In addition, children who communicate about various issues with their parents not only have satisfaction communicating with the parents; they also perceive relational satisfaction (Schrodt, & Afifi, 2007). Therefore, children who frequently communicate with their parents may sense parents’ affection and care, and they may perceive relatively more communication satisfaction than those who rarely communicate with their parents. In line with the individuation and connectedness perspective, communication satisfaction, which signifies positive parent-child climate, might negate damaging peer influence, and adolescents may perpetrate behaviors that are in line with values that parents teach (i.e., making good friends and avoiding delinquent behaviors). Hence, we posed a hypothesis to look at the mediating role of peer influence between communication satisfaction and adolescents’ alcohol use:

*H4*: Peer influence will mediate the association between parent-child communication satisfaction and Korean adolescents’ alcohol use.

## 2. Method

### 2.1 Participants and Procedures

Five-hundred thirty-eight adolescents (363 male, 174 female) from four high schools in the Seoul metropolitan area in Korea participated in the current study. To provide representation from the different districts, four high schools were selected. Male participants were recruited from four high schools (n = 134, 91, 73, 65), and female participants were recruited from one of the high schools (n = 174). Because drinking is problematic among boys in Korea, most of the high school principals authorized male students to be the sample for the current study. As a result, male students were recruited from all four schools and female students were recruited from one school. Respondents’ ages ranged from 14 to 17 years, and their mean age was 15.32 years (*SD* = 1.02).

Homeroom teachers announced the current study in their classroom and asked class leaders to administer survey procedures. When the teachers exited the classroom, the class leaders distributed paper survey booklets to the students. Students were informed that completing and returning a survey packet was entirely voluntary. To reduce obtrusiveness, only written directions were provided, and the class leaders did not interact with the students. The survey booklets themselves were anonymous as no personally identifiable information was collected. Researchers obtained institutional approval to collect the data in high schools.

### 2.2 Measurements

The measures used in the current study were translated into Korean by a researcher. Then, the translations were back-translated into English by a bilingual translator blind to the original English version. Next, the back-translated version was checked for consistency with the original. The back-translated version closely matched the original English version.

Wood *et al*.’s (2004) parental monitoring scale was used for the current study. This scale was based on Steinberg, Lamborn, Dornbusch, and Darling’s (1992) strictness-supervision scale. Three items asked respondents what their parents actually know and what their parents attempt to know about their behaviors. Specifically, an example question read “How much do your parents try to know and (really know) about what you do with your free time?” Each item was answered with the following options: 1 = don’t try or don’t know, 2 = try a little or know a little and 3 = try a lot or know a lot. The *alpha* coefficient of the parental monitoring scale was .88 (*M* = 2.50, *SD* = .45).

Parent-child communication satisfaction was measured with a modified version of Hecht’s (1978) interpersonal communication satisfaction questionnaire. Ten items asked how respondents generally describe their communication behavior with their father, and another set of identical questions asked about their communication behavior with their mother. An example questions include “I was very dissatisfied with conversations with him/her”. Each item was followed by a 5-point Likert-type scale with 1 representing “strongly disagree” and 5 representing “strongly agree.” The *alpha* coefficients of the father-child communication satisfaction scale was .88 (*M* = 3.29, *SD* = .76), and the mother-child communication satisfaction scale was .86 (*M* = 3.65, *SD* = .68). In the structural model, parent-child communication satisfaction was a latent variable with father-child and mother-child communication satisfactions subscales.

Peer influence was measured with a modified version of Williams *et al*.’s (1995) scale. The items for the scale were adopted from the research of Donovan, Costa, and Jessor (1985), Johnston, O’Malley, and Bachman, (1989), and Oetting, Beauvais, Edwards, Edwards, and Waters (1984). The peer influence scale asked how often the respondents’ friends have asked them to (a) smoke cigarettes, (b) drink alcohol, and (c) get drunk. Each item was followed by a 5-point Likert-type scale with 1 representing “never” and 5 representing “many times.” Since marijuana, smokeless tobacco, and cocaine are not accessible in Korea, the items concerning these types of drugs were not used in the present study. The *alpha* coefficient of the peer influence scale was .86 (*M* = 1.84, *SD* = .98).

Respondents’ prior alcohol use was measured by a past alcohol use subscale from Perry and Grant’s (1988) alcohol use tendency scale. To assess respondents’ past alcohol use, four items asked how many occasions they have had alcoholic beverages to drink (a) during the last 12 months, (b) during the last 30 days, and (c) during the last 7 days. Each item was answered with the following options: 0 = never; 1 = 1 - 2 occasions; 2 = 3 - 5 occasions; 3 = 6 - 10 occasions; 4 = 11 - 20 occasions; 5 = 21 - 39 occasions; 6 = 40 or more occasions. The three-item prior drinking measure had the *alpha* coefficient of .85 (*M* = .64, *SD* = .91). The mean score indicates that respondents, on average, had no more than 2 instances of drinking events in the last year.

To assess respondents’ drinking refusal self-efficacy, a modified version of Perry and Grant’s (1988) drinking refusal self-efficacy scale was selected for the current study. The items asked how sure respondents were that they could say no if they were offered alcohol (a) at a friend’s house, (b) by an older brother or sister, (c) by other older persons, and (d) at a party or dance. One question from the original scale that asked if respondents could say no when their boyfriend/girlfriend offered alcohol was deleted for the current study because dating in high school is atypical in the Korean culture. Each item was followed by a 5-point Likert-type scale with 1 representing “could say no” and 5 representing “could not say no.” The items were recoded so that higher scores represented high drinking refusal self-efficacy. The *alpha* coefficient of the scale was .90 (*M* = 3.24, *SD* = 1.14).

Drinking intentions was measured with a modified version of an alcohol use prospect scale (Perry & Grant, 1988). The scale asked how likely it would be that they would drink an alcoholic beverage if someone offered it to them (a) in the next 12 months, (b) in the next 30 days, and (c) in the next 7 days. Each item was followed by a 5-point Likert-type scale with 1 representing “likely I would not drink” and 5 representing “likely I would drink.” The *alpha* coefficient of the scale was .88 (*M* = 2.63, *SD* = 1.06).

## 3. Results

**Figure 1:**
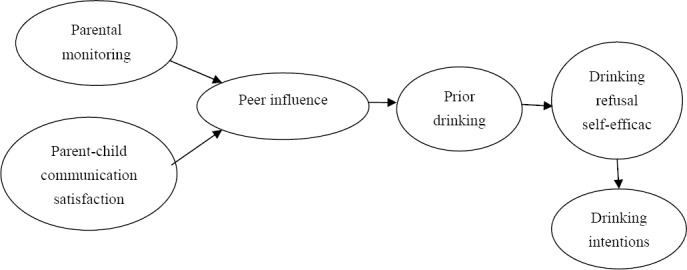
Hypothesized Model

The hypotheses were tested by Structural Equation Modeling. The model was built with Amos 18. This procedure was appropriate because structural equation modeling was able to clarify the direct and indirect associations in the test of multivariate hypotheses. Our model was developed by constructing the paths predicted by our hypotheses (please see Figure 1). Specifically, parental monitoring and parent-child communication satisfaction were the two exogenous variables predicting peer influence. Peer influence, prior drinking, and drinking refusal self-efficacy were antecedent endogenous variables, with the first predicting prior drinking, the second predicting drinking refusal self-efficacy, and the latter predicting drinking intentions. Finally, drinking intentions was the outcome endogenous variable.

All the variables were operationalized as latent variables, since the latent composite approach could “account for unreliability by extracting measurement error from the latent constructs used in the structural model” (Holbert & Stephenson, 2002, p. 534). Both direct and indirect effects of the related variables were calculated. A bootstrap for each model (number of bootstrap samples is 2000) was performed, and 95% bias-corrected confidence intervals were used to test the significance of the direct and mediation effects. To gauge the fit of the structural equation models, an omnibus model fit was evaluated using the comparative fit index (CFI) and the root mean squared error of approximation (RMSEA). Prior criteria we used were .90 for CFI and .08 for RMSEA. In addition, given the guidelines of Hoyle and Panter (1995), the chi-squared distributed goodness of fit test was also reported. Results of the analysis revealed that our model did not meet the established priori criteria, χ^2^ (*df* = 14, *N* = 537) = 359.57, *p* < .001 CMIN/*df* = 25.68, CFI = .69, RMSEA = .21.

Subsequently, we made modifications in the model. First we removed paths one by one based on the Lagrange multiplier test, and afterward, we inserted paths one at a time based on the Wald’s test (see Knobloch, Solomon, & Cruz, 2001, for an overview of this procedure). We eliminated one path from the proposed model as a result of the Lagrange multiplier test (Fox, 1997): The path from parent-child communication satisfaction to peer influence. Because eliminating the path made parent-child communication satisfaction variable unidentifiable (i.e., there was no path assigned from or to this variable), this model could not be tested. Then, we added paths to the model using the Wald’s test (Fox, 1997). When adding paths, we were very careful to follow previous research. Based on the findings of previous work that suggest a positive link between parent-child communication and parental monitoring (Stattin & Kerr, 2000), we examined the association. In view of that, a path from parent-child communication satisfaction to parental monitoring was added.

Two additional paths were added in the model: A path from parental monitoring to drinking refusal self-efficacy and another path from peer influence to drinking refusal self-efficacy. Watkins *et al*. (2006) suggest that parental monitoring is positively associated with adolescents’ drinking refusal self-efficacy. Adolescents who believe they receive a lot of parental monitoring also believe that they have high drinking refusal self-efficacy. Nash *et al*. (2005) similarly suggest that family environment, which includes parental monitoring, is positively associated with adolescents’ drinking refusal self-efficacy. Based on the findings, a path from parental monitoring to drinking refusal self-efficacy was added. Next, Young, Hasking, Oei, and Loveday (2007) argue that “the role of refusal self-efficacy in the development and maintenance of drinking behavior, including in situations of *peer pressure*, is well established” (p. 863). Given that the drinking refusal self-efficacy measure reflects peer pressure refusal self-efficacy, it is evident that peer pressure may sway individuals’ drinking refusal self-efficacy (Young & Oei, 2000). Thus, we added another path from peer pressure to drinking refusal self-efficacy. Finally, based on the previous research that suggest a strong association between prior drinking and drinking intentions (Aas *et al.*, 1995), a path from past drinking and drinking intentions was added.

**Figure 2:**
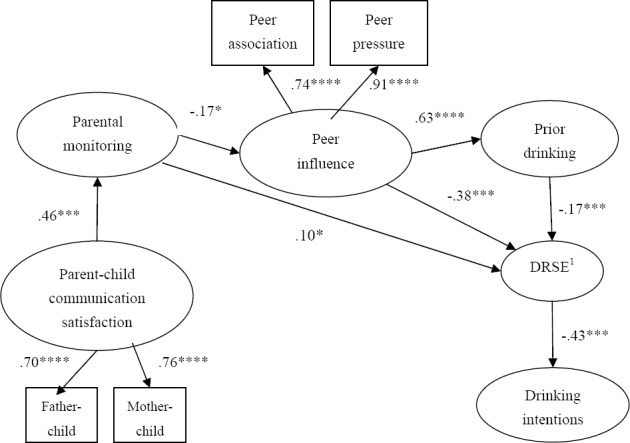
Revised Structural Model *Note*: ^1^ Drinking refusal self-efficacy. All parameter estimates are standardized. * *p* < .05, *** *p* < .001

After we made these modifications, the revised model was consistent with the data and established priori criteria, χ^2^ (*df* = 11, *N* = 537) = 15.40, *p* = .17, CMIN/*df* = 1.40, CFI = .996, RMSEA = .027. Please see Figure 2 for the revised model. The direct effect of drinking refusal self-efficacy on drinking intentions (*H1*) (β = - .43, *p* < .001) and past drinking on drinking refusal self-efficacy (*H2*) (β = - .23, *p* < .001) were significant. Thus, *H1* and *H2* were supported. The indirect effect of parental monitoring on drinking behavior, mediated by peer influence was also significant (*H3*) (standardized mediation effect = - .09, *p* < .01). The Sobel mediation test was also conducted to examine the mediating role of peer influence between parental monitoring and drinking behavior. The analysis showed that the indirect effect of parental monitoring on adolescents’ drinking was significant (*z* = - 3.32, *p* = .001). Accordingly, *H3* was supported by the current data. We were unable to fully examine *H4* since the link between parent-child communication satisfaction and peer influence was removed during the initial stage of the analyses. The revised model, however, suggests that parent-child communication satisfaction is positively associated with parental monitoring (β = .46, *p* < .001). Thus, *H4* was not supported. Finally, the total indirect effect of parent-child communication satisfaction on adolescents’ drinking intentions, mediated by parental monitoring, peer influence, prior drinking, and drinking refusal self-efficacy, was significant (standardized mediation effect = - .03, *p* < .01).

## 4. Discussion

The current study examined the function of parental influence, peer influence, and prior drinking on Korean high school students’ drinking refusal self-efficacy. The revised model revealed that a number of factors directly influence Korean adolescents’ drinking refusal self-efficacy, including prior drinking, parental monitoring, and peer influence. Consistent with social cognitive theory (Bandura, 1986), adolescents’ drinking experience was a factor that predicts their self-efficacy regarding drinking refusal. Adolescents who had consumed alcohol in the past reported that they have relatively low drinking refusal self-efficacy compared to abstinent counterparts. In addition, the results of this study extend previous work by indicating that parental factors, including parent-child communication satisfaction and parental monitoring, are uniquely linked to adolescents’ alcohol use through peer influence. And, adolescents’ drinking refusal self-efficacy was a mediator between prior drinking and their intentions to consume alcohol in the future. The discussion below will highlight the findings with regard to the revised structural model and discuss the present findings in terms of previous research.

### 4.1 Interpretations of the Findings

In order to fully identify the factors that influence adolescents’ drinking refusal self-efficacy, we first sought to understand what motivated those adolescents with prior alcohol experience to initiate drinking. Based on a number of theories (i.e., developmental theory, socialization theory, individuation-connectedness perspective) that suggest a mediating role of peer influence between parental influence and children’s drinking (Patterson *et al.*, 1989; Steinberg, 2001; Wood *et al.*, 2004), we examined the indirect effect of parental influence on adolescents’ drinking through peer influence. Indeed, findings of the current study revealed that parental monitoring is associated with peer influence, and peer influence, in turn, is related to adolescents’ alcohol use. In line with the notion of socialization theory, parents could protect their children from peer influence by monitoring their activities and whereabouts outside the home (Grusec, & Davidov, 2010). By restricting children’s contacts with problem peers, parental monitoring can prevent adolescents’ participation in underage drinking (Westling *et al.*, 2008). The findings also imply that adolescents who receive relatively little parental monitoring are susceptible to greater peer influence, which directly impacts their alcohol use. The finding also supports previous research that demonstrates the positive association between peer influence and adolescents’ drinking (Hwang & Akers, 2006; Wood *et al.*, 2004).

However, parent-child communication satisfaction does not have an effect similar to that of parental monitoring on peer influence. Instead, parent-child communication satisfaction facilitates parental monitoring. In agreement with this result is Stattin and Kerr’s (2000) findings that highlight the effects of good parent-child communication on parental monitoring practices. Our data indicated that parent-child communication satisfaction was another contributor to Korean adolescents’ perceived parental monitoring. Because adolescents are likely to share information about their whereabouts and activities outside the home during communication with the parents, those adolescents who are content communicating with their parents are more likely to believe that their parents know a lot about their daily activities. Consistent with this idea, Cohen and Rice (1995) found that parent monitoring and maintaining communication with parents protected adolescents from substance use.

In addition to the effects of Korean adolescents’ prior drinking on their drinking refusal self-efficacy, the present research revealed that parental monitoring and peer influence directly influence their drinking refusal self-efficacy. According to Watkins *et al*. (2006), a high level of parental monitoring may allow adolescents to believe that they can refuse drinking alcohol. Another study that supports the idea is Nash *et al*.’s (2005) study that reports family environment, which includes parental monitoring, is positively associated adolescents’ drinking refusal self-efficacy. Nash *et al*. suggest that peer influence mediates the link between family influence and alcohol use; however, they are mute about the link between peer influence and drinking refusal self-efficacy. Although these findings are conclusive, explanations for the effects of parental monitoring and peer influence on drinking refusal self-efficacy are incomplete. The results of the current study are consistent with social cognitive theory (Bandura, 1986), which suggests the importance of environment (i.e., family and friends) on shaping individuals’ self-efficacy perceptions. Our respondents who had high level of parental monitoring had relatively little peer pressure, and they also believed they had a relatively high drinking refusal self-efficacy. By contrast, those who reported having little parental monitoring also reported having relatively more peer pressure, and they perceived having a relatively low drinking refusal self-efficacy. Consistent with Steinberg’s (2001) argument, our findings show that high level of parental monitoring could not only negate peer influence, it could also boost children’s self-efficacy. In a similar vein, peer influence may predict adolescents’ drinking refusal self-efficacy. Adolescents who perceive little parental monitoring are in a vulnerable condition for peer influence. Social cognitive theory (Bandura, 1986) suggests that one method to increase self-efficacy is to be with people who are positive about and successful in achieving goals and outcomes. Close friends’ alcohol use may influence adolescents’ internalization of the behavior and increase confidence in their own ability to drink alcohol themselves. As a result, Korean adolescents who are heavily influenced by their peers may perceive low drinking refusal self-efficacy.

It is also important to note that Korean adolescents’ prior drinking not only influences their drinking intentions indirectly through drinking refusal self-efficacy, it also directly affects their drinking intentions. Korean adolescents with drinking experience are more likely to have intentions to use alcohol in the future when compared with the abstinent counterparts. The result is consistent with previous research that suggests past substance use can be a good predictor of future substance use (Patrick, Wray-Lake, Finlay, & Maggs, 2010). Eagly and Chaiken (1993) explain that because behavior over time is the result of people’s personal and motivational attributes that are common to the events in which the behavior occurs, they generally act consistently.

### 4.2 Implications

Taken as a whole, our findings add to the literature that emphasizes the importance of adolescents’ drinking refusal self-efficacy in predicting their drinking intentions. Specifically, our data indicated that parental monitoring, peer influence, and prior alcohol use are uniquely associated with adolescents’ drinking refusal self-efficacy, and drinking refusal self-efficacy, in turn, predicts their drinking intentions. These findings support Bandura’s claim that successful experience predicts self-efficacy, and self-efficacy predicts future behavior. Bandura (1986) also suggest that environment affects people’s behavior and attitude. Consistent with this idea, our findings demonstrate that when adolescents are in an environment where the parents closely watch their behaviors and are influenced less by peers, they may perceive relatively high self-efficacy to refuse drinking. The findings considerably add to the literature on antecedents of adolescents’ drinking refusal self-efficacy as research on this area is limited.

Another notable contribution of the current study involves our use of a Korean high school sample. As influenced by Confucianism, Asian families or families in a collectivistic culture tend to follow a traditional family structure, characterized by a high degree of cohesiveness and hierarchy (Yum, 1988). In traditional Asian families, parents make the decisions for the family, and children are expected to respect these decisions with compliance (Hofstede, 1991). As a consequence, parental authority is often viewed as customary. Rohner and Pettengill (1985) suggest that Korean high school students’ perceived parental control is positively associated with parental warmth and caring. Although adolescents in Western cultures may view parental strictness as aggressive (Rohner & Pettengill, 1985), Korean adolescents may interpret strict parental monitoring as an expression of parental warmth and caring. Therefore, the influence of parents may be more distinct and seen as positive in Eastern cultures than in Western cultures. This may explain the parental influence we found in the current study.

There are a number of practical implications based on this investigation. It is thought that children are heavily influenced by their peers’ deviant behaviors (Monahan, Steinberg, & Cauffman, 2009). Although peer influence may be an important predictor of adolescents’ drinking behavior, we found evidence of the effects of parental influence on children’s drinking. Based on the findings of this research, alcohol prevention interventions need to keep considering parents in the overall strategy. The message is clear: Parents should get actively involved to reduce children’s alcohol use by engaging in communication with children to monitor their alcohol-related behaviors. These efforts may influence their adolescent children’s drinking refusal self-efficacy perception, and may ultimately decrease children’s drinking. We learned that parent-child communication satisfaction is an effective way to show parental monitoring. Children who are satisfied conversing regularly with their parents also believed that they are closely observed by their parents.

### 4.3 Limitations

A number of limitations of the current research must be recognized. One of these involves the use of retrospective self-reports. Participants may not have accurately recalled or reported their attitudes and behavior (Bernstein, Erdfelder, Meltzoff, Peria, & Loftus, 2011). Since asking adolescents directly about their intentions to drink alcohol is a simple and sensible way to evaluate their intentions, we utilized this method. Another limitation of the present study is the sample. The current sample is the 2 to 1 ratio of males to females. While independent sample t-tests of the study variables suggest that none of the variables exposed sex differences, this sample bias may limit the ability to generalize the results. Further, because the influence of parents may be more marked in Eastern than in Western cultures (Hofstede, 1991), findings of this study may be less generalizable to Western cultures. In addition, we certainly understand that the present investigation is unable to determine the causal directions of the aforementioned effects. More research needs to be done to fully understand the effects of parents and peers on adolescents’ drinking refusal self-efficacy and drinking intentions.

## 5. Conclusion

In conclusion, the main purpose of the present study was to identify the factors that influence Korean adolescents’ drinking refusal self-efficacy. Findings revealed that parental monitoring and peer influence directly and indirectly predicted drinking refusal self-efficacy through adolescents’ alcohol use. We identified the impact of parents and peers on Korean adolescents’ drinking. Furthermore, this study revealed that those factors are antecedents of the adolescents’ drinking refusal self-efficacy, which, in turn, predicts their drinking intentions. Given the results of our study, we are convinced that these constructs should be the key attributes researchers should consider when studying Korean adolescents’ alcohol use.

**Table 1:** *Zero-Order Correlation Matrix of the Study Variables*.

	1	2	3	4	5	6	7
1.1. Parental monitoring	--						
2. Father-child comm. satisfaction	.31***	--					
3. Mother-child comm. satisfaction	.35***	.68***	--				
4. Peer influence	-.16***	-.08	-.08	--			
5. Past alcohol use	-.17***	-.09*	-.11**	.57***	--		
6. Drinking refusal self-efficacy	.19***	.14***	.13**	-.46***	-.42***	--	
7. Drinking intentions	-.18***	-.14***	-.14***	.49***	.66***	-.63***	

*Note*.

* *p* < .05,

** *p* < .01,

*** *p* < .001
